# Do Flavonoids from Durum Wheat Contribute to Its Bioactive Properties? A Prospective Study

**DOI:** 10.3390/molecules26020463

**Published:** 2021-01-17

**Authors:** Adriano Costa de Camargo, Anna Paula de Souza Silva, Jackeline Cintra Soares, Severino Matias de Alencar, Cíntia Ladeira Handa, Karina Silva Cordeiro, Marcela Souza Figueira, Geni R. Sampaio, Elizabeth A. F. S. Torres, Fereidoon Shahidi, Andrés R. Schwember

**Affiliations:** 1 Laboratory of Antioxidants, Nutrition and Food Technology Institute, University of Chile, Santiago 7830490, Chile; 2 Departamento de Ciencias Vegetales, Facultad de Agronomía e Ingeniería Forestal, Pontificia Universidad Católica de Chile, Santiago 7830490, Chile; 3 Departament of Agri-Food Industry, Food & Nutrition, “Luiz de Queiroz” College of Agriculture, University of São Paulo, P.O. Box 9, Piracicaba, SP CEP 13418-900, Brazil; anna.paula.silva@usp.br (A.P.d.S.S.); jacksoares27@hotmail.com (J.C.S.); smalencar@usp.br (S.M.d.A.); 4 Minas Gerais State University, R. Ver. Geraldo Moisés da Silva 308-434, Ituiutaba, MG CEP 38302-182, Brazil; cintiahanda@gmail.com; 5 Department of Nutrition, School of Public Health, University of São Paulo, 715 Dr. Arnaldo Avenue, São Paulo, SP CEP 01246-904, Brazil; karinacordeiro@usp.br (K.S.C.); msfigueira@usp.br (M.S.F.); genirs@usp.br (G.R.S.); eatorres@usp.br (E.A.F.S.T.); 6 Department of Biochemistry, Memorial University of Newfoundland, St. John’s, NL A1B 3X9, Canada; fshahidi@mun.ca

**Keywords:** *Triticum turgidum* L. var. *durum*, surface response methodology, LC–ESI-QTOF-MS, antiradical activity, pancreatic lipase, obesity

## Abstract

A clear gap with respect to the potential biological properties of wheat flavonoids exists in the available literature. This information is crucial for breeding programs aiming to produce new varieties presenting improved health benefits. Accordingly, advanced breeding lines of whole durum wheat were evaluated in this contribution. The highest recovery of phenolics was achieved using aqueous acetone (50:50, *v/v*), as verified by multi-response optimization, thus showing that phenolics could be largely underestimated by employing an inappropriate extraction. The concentration of derivatives of apigenin, the main phenolics present, ranged from 63.5 to 80.7%, as evaluated by LC–ESI-QTOF-MS. Phenolics from the breeding line 98 exhibited the highest ability in scavenging peroxyl radicals, reducing power as well as in terms of inhibition of pancreatic lipase activity, a key enzyme regulating the absorption of triacylglycerols. In contrast, none of the samples exhibited a significant anti-diabetic potential. Despite their high concentration compared to that of phenolic acids, results of this work do not support a significant antioxidant and pancreatic lipase inhibitory effect of durum wheat flavonoids. Therefore, breeding programs and animal and/or human trials related to the effect of durum wheat flavonoids on oxidative stress and absorption of triacylglycerols are discouraged at this point.

## 1. Introduction

Wheat is a major crop worldwide, belonging to the Poacea (grasses) botanical family, which serves as the staple food for many populations [1]. Durum wheat (*Triticum turgidum* L. var. *durum*) is the main component of various foods such as bread, pasta, and cakes [2,3,4,5]. Besides pasta, it may also be used in the manufacture of bulgur, and couscous, among others [5,6,7].

Like other cereals and their processing by-products, wheat is a known source of phenolic acids [1,2] although other bioactive compounds such as bioactive peptides have been reported [3]. In plants, phenolic compounds perform important functions that include protection against the infection caused by microorganism and insect depredation. Excessive production of reactive oxygen species (ROS) may lead to plant cell death [4]. Phenolics are modulated by environmental conditions, including drought tolerance stress. These molecules also protect the plant against oxidative stresses derived from ultraviolet radiation and high intensity light [5,6]. Likewise, the phenolic content has been found to change during rainfed condition. Therefore, phenolic antioxidants are modulated during drought tolerance [7,8].

Humans also benefit from the consumption of plant foods due to their richness in bioactive compounds [9,10,11], which render antioxidant, anti-inflammatory, antimicrobial as well as sensorial properties [12,13,14,15]. The role of phenolic compounds in protection against the development of cardiovascular diseases and certain types of cancer has been well documented [5,16,17,18]. In addition, in recent years, there are increasing evidence suggesting their role in the management and/or prevention of weight gain and obesity [19], among other potential health benefits [20].

Many efforts have been made in unrevealing the digestion and absorption of phenolic compounds [21,22], all of which help in better understanding their systemic effects as non-metabolized and/or non-absorbed phenolics in exerting their effects locally upon gastric digestion [2]. Furthermore, lipase and alpha-glucosidase are involved in the digestion of triacylglycerols and carbohydrates, respectively [19,23]. Therefore, the inhibitory effect of phenolic compounds toward enzymes related to obesity and type 2 diabetes does not depend solely on their absorption.

Due to the availability of modern identification tools (e.g., hyphenated techniques including high resolution mass spectrometry analysis) some authors have been able to move beyond the identification of phenolic acids, also addressing the presence of monomeric flavonoids as aglycones as well as in their conjugated form. However, only a few research groups [24,25,26,27] have investigated the antioxidant properties of the feedstocks by different methods but none of them have specifically addressed the possible contribution of derivatives of apigenin derivatives, although these compounds were reported many times [24,25,28,29]. Likewise, none of the mentioned reports have studied the potential of phenolics from wheat in inhibiting the activity of alpha-glucosidase and pancreatic lipase. Therefore, a clear gap with respect to relative biological properties of durum wheat currently exists in the available literature.

Considering the lack of literature clarifying if whether flavonoids could significantly contribute or not to the potential bioactive properties of durum wheat, advanced breeding lines were evaluated for their phenolic profile by high definition accurate-mass spectrometry (LC–ESI-QTOF-MS), antiradical activity, reducing power and inhibitory effects toward pancreatic lipase and alpha-glucosidase. Therefore, the present contribution advances the knowledge concerning the potential bioactivity of durum wheat flavonoids. This is deemed necessary, especially in wheat breeding programs that must define their quality traits. Furthermore, our results may also guide epidemiological studies aiming to correlate the intake of wheat phenolics, including phenolic acids and flavonoids, and the potential health outcomes.

## 2. Results and Discussion

### 2.1. Multi-Response Optimization of the Effects of Solvent System in Relation to TPC

Statistics combined with surface response methodology (RSM) have been employed in food science and technology as well as in the recovery of phytochemicals [30]. In fact, mixture designs (e.g., simplex-centroid) facilitate the study of synergistic and/or antagonistic effects of solvent combinations in order to obtain the highest extraction of phenolic compounds [31]. The application of RSM decreases the number of combinations or experiments but it requires a careful statistical evaluation for further validation [32,33].

The correlation of total phenolics with antiradical activity and reducing power of many feedstock and by-products thereof is well documented [34,35,36]. Likewise, positive correlations between total phenolic content (TPC) and the inhibition of digestive enzymes (e.g., alpha-glucosidase and pancreatic lipase) have also been reported [37]. Therefore, TPC was chosen parameter to be optimized. The influence of three solvents on the extraction of phenolic compounds using a simplex-centroid mixture design is shown in Table 1, whereas the coefficient of determination (R^2^) and the F test (analysis of variance-ANOVA) are shown in Table 2.

The highest TPC was found using aqueous acetone (50%, *v/v*) for both breeding lines. In contrast, regardless of the option, single solvents always showed lower extraction efficiency compared to that of their mixtures in water. The TPC of the extract obtained using aqueous acetone (50%, *v/v*) increased by up to 6.8 times in comparison to acetone itself. In addition, the TPC of the sample subjected to extraction in acetone in water (50%, *v/v*) was up to 3.6-fold higher than that of the extract obtained using methanol and up to 38% higher compared to that of methanol in water (50%, *v/v*). Therefore, the results showed that the TPC varied greatly among different solvent systems.

All the independent and response variables were fitted to a special cubic model. The predicted regression equations represent the models with the significant factors for TPC from breeding line 98 (y1) and breeding line 5 (y2) were:

(1) $$y1=28.61x_{1}+77.18x_{2\;}+54.94x_{3}+461.99x_{1}x_{2}+226.55x_{2}x_{3}$$

(2) $$y2=23.59x_{1}+73.99x_{2\;}+38.86x_{3}+341.78x_{1}x_{2}+191.43x_{2}x_{3}+519.19x_{1}x_{2}x_{3}$$

**Figure 1:**
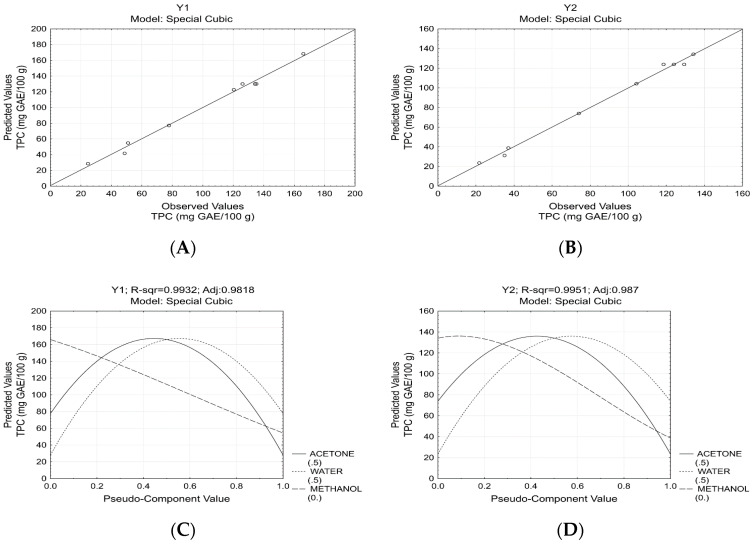
Plots of the observed versus the predicted and plots of estimated responses. (**A**): Plot of the observed versus the predicted for total phenolic content (TPC) from Breeding Line 98 (y1); (**B**): Plot of the observed versus the predicted for TPC from Breeding Line 5 (y2); (**C**): Plot of estimated response for y1; (**D**): Plot of estimated response for y2.

The coefficient of determination (*R*^2^), the F test (analysis of variance-ANOVA), and the plots of the observed versus the predicted responses were used to verify the quality of fit of the models Table 2; Figure 1A,B.

The high *R*^2^ (both above 0.99), the absence of lack of fit as well as the calculated F-value of the models, which were greater than the F tabulated (6.39 and 9.01, respectively for y1 and y2) indicating that all response functions adequately fit the experimental data. Therefore, regardless of the breeding line, the models can be employed for predictive objectives. The plots of the predicted relative values to the observed responses, indicating that the experimental points were related, reflecting their normal distribution, visually confirming the model quality Figure 1A,B.

**Figure 2:**
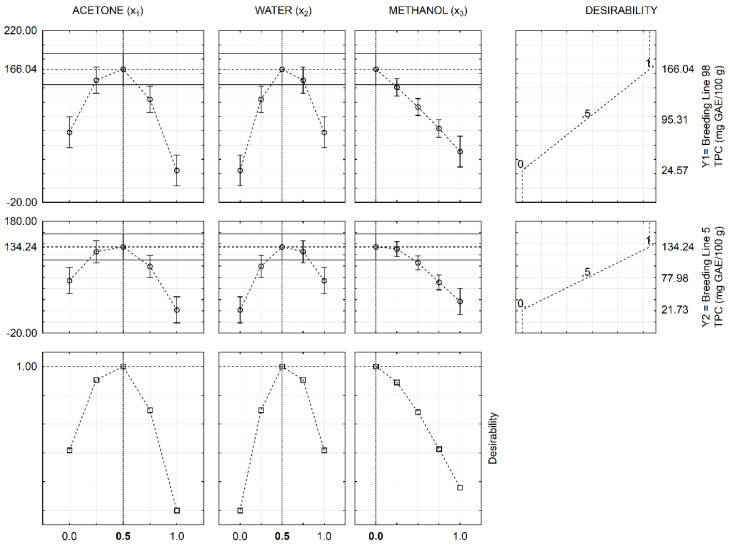
Profiles for the predicted values and overall desirability as a function of the solvent system used for extraction of phenolic compounds.

The positive linear terms showed that all solvents can be used for extraction of phenolic compounds Equations (2) and (3). In addition, the positive quadratic and cubic terms indicated synergistic effects of the solvent mixtures on the TPC. As observed in both response functions, the binary mixture of acetone/methanol does not have a significant effect and was excluded from the models. In contrast, the binary mixtures containing water induced the greatest effect on the extraction of phenolic compounds, with the water and acetone mixture making the highest contribution to the model compared to water and methanol mixture. This fact can also be observed in Figure 1C,D, at which the plots of estimated responses indicated that higher TPC were observed when the methanol is near to zero and water and acetone is about 0.5:0.5 (*v/v*, acetone/water) or 50% aqueous acetone. In addition, we can verify that pure solvents are not appropriate in terms of TPC since all of them reduced the response. Finally, according to the joint optimization of the TPC from breeding lines 98 and 5 Figure 2, the maximum responses were estimated at solvent ratios of 0.5:0.5:0.0 (*v/v/v*, acetone/water/methanol), confirming what was reported earlier in the present study Figure 1C,D.

Therefore, by using the optimal solvent mixture, acetone/water (0.5:0.5, *v/v*), the predicted values (y_1_ = 166.04 ± 30.53 mg GAE/100 g and y_2_ = 134.44 ± 23.20 mg GAE/100 g) agree with the experimental values, therefore also supporting the predictive role of the models. This result is interesting since the boiling point of acetone is lower than that of methanol (56.0 °C and 64.7 °C, respectively) [38], which facilitates solvent evaporation. This is important for the application of phenolic compounds to other matrices, such as food, cosmetics, or drugs as well as to easily remove the organic solvent in experiments involving fractionation techniques (e.g., free, esterified and etherified fractions). In addition, methanol shows higher toxicity than acetone. Therefore, our contribution is also helpful to decrease human exposure during extraction procedures. Wheat products possess phenolic antioxidants bearing different levels of polarity [38], thus supporting the different extraction yields obtained in the present study. As shown by Handa et al. [31], the mixture design is an important statistical tool, being effective in clarifying the interaction between the solvent and the test material and the outcome in terms of recovery of phenolic compounds.

Phenolic quantification is highly influenced by sample preparation. Literature data on quantification of phenolic compounds has been used to create the USDA phenolic databases [39,40,41]. These tools provide important data for studies aiming to assess the health and nutritional status of adults and children such as the National Health and Nutrition Examination Survey (NHANES), a program of the Center for Disease Control and Prevention (CDC). Therefore, to avoid phenolic underestimation that may jeopardize any conclusion on the relation of phenolic intake versus health outcome, especially in epidemiological studies, our study indicates that aqueous acetone (50%, *v/v*) should be used instead of aqueous methanol or aqueous ethanol, the most frequently employed thus far.

### 2.2. Phenolic Profile

**Figure 3:**
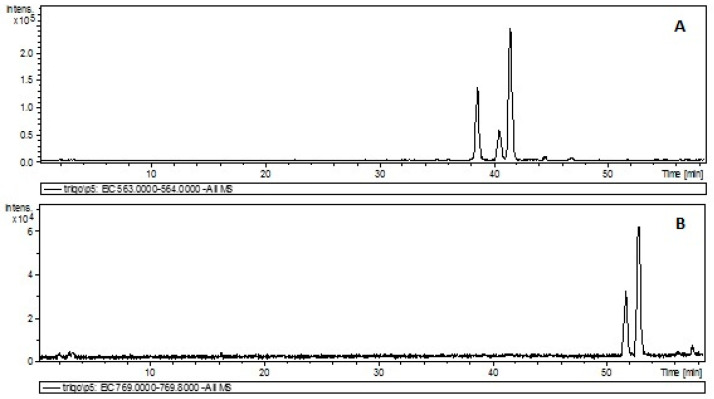
HPLC–ESI-QTOF-MS base peak chromatogram (BPC-All MS) of durum wheat soluble extracts (Breeding Line 5) obtained under optimized conditions (**A**) apigenin-6-C-arabinoside-8-C-hexoside (**B**) apigenin-6-C-beta-galactosyl-8-C-beta-glucosyl-*O*-glucuronopyranoside.

The compounds *p*-coumaric and ferulic acids were identified by comparison with coded and authentic standards under the same conditions as the samples Table 3. Pinosylvin (double glycosylation) was putatively identified due to its deprotonated molecular ion at 535.1813 calculated error (1.4948 ppb) compared to that of the literature [25,28]. Different isomers exhibiting [M-H]^−^ ranging from 563.1395 to 563.1406 have been reported [24,25,28,29]. Considering these authors, the molecule presenting *m/z* of 563.1400 and error ranging from 4.2542 to 4.6087 ppb, depending on the author, was putatively identified as apigenin-6-C-arabinoside-8-C-hexoside. Likewise, apigenin-6-C-beta-galactosyl-8-C-beta-glucosyl-*O*-glucuronopyranoside isomers, [M-H]^−^ at 769.1977 *m/z*, was putatively identified based on the cited literature [28,29]. Figure 3A brings about the base peak chromatogram (BPC) of apigenin-6-C-arabinoside-8-C-hexoside, lending support to the presence of three isomeric forms, while Figure 3B shows BPC of apigenin-6-C-beta-galactosyl-8-C-beta-glucosyl-*O*-glucuronopyranoside, also substantiating the presence of two isomers. Finally, the experimental errors mentioned here were lower than those of authentic standards (5.4085 to 9.1437 ppb), which were evaluated using the same equipment for comparison purposes in order to validate the method employed.

More than 8000 (poly)phenols have already been identified. However, only a few authentic standards are commercially available [42]. Therefore, although important for a first screening, any study based on HPLC itself, which requires phenolic standards for comparison of their retention times, and UV spectra, is considered to be a partial identification. The use of liquid chromatography coupled to low-resolution mass spectrometry (e.g., HPLC–DAD–ESI–MS^n^) [43] gives better results because, in addition to UV spectral data, the identification is made based on the same ionization pattern considering the available standards as well as the literature data [43,44]. However, as demonstrated by Marczak et al. [45], ion chromatograms obtained by employing high resolution mass spectrometry analysis are able to provide *m/z* values presenting a very high mass accuracy (±0.005 mass unit), thus allowing the putative identification of compounds bearing the same nominal masses.

Regardless of the feedstock, apigenin derivatives were the main phenolics quantified by LC–ESI-QTOF-MS. The concentration of derivatives of apigenin (e.g., apigenin-6-C-arabinoside-8-C-hexoside and apigenin-6-C-beta-galactosyl-8-C-beta-glucosyl-*O*-glucuronopyranoside) ranged from 63.5 to 80.7%, while the contribution of phenolic acids was much lower (19.3%). Ferulic acid was the main phenolic acid in both samples Table 4, which is supported by previous studies [28,46]. It has been reported that generally, the absorption of phenolic acids is very rapid between 1–2 h after food intake [21]. As aforementioned, different isomers of pinosylvin (double glycosylation), apigenin-6-C-arabinoside-8-C-hexoside, and apigenin-6-C-beta-galactosyl-8-C-beta-glucosyl-*O*-glucuronopyranoside were identified due to their different retentions times, which agrees with the available literature [28]. However, to simplify the situation, Table 4 shows the total concentration of each one of them.

**Table 3:** Phenolic profile of whole durum wheat as evaluated by LC–ESI-QTOF-MS.

Compounds	Rt * (min)	Molecular Formula	[M-H]^−^	Breeding Line 5	Breeding Line 98	Reference
feruloylquinic acid	26.6	C_17_H_20_O_9_	367.1018	+	−	[47]
*p*-coumaric acid	36.7	C_9_H_8_O_3_	163.0402	−	+	
ferulic acd	41.3	C_10_H_10_O_4_	193.0516	+	+	[47]
apigenin-6-C-arabinoside-8-C-hexoside (isomer 1)	38.6	C_26_H_28_O_14_	563.1426	+	+	[28,29]
apigenin-6-C-arabinoside-8-C-hexoside (isomer 2)	40.5	C_26_H_28_O_14_	563.1428	+	+	[28,29]
apigenin-6-C-arabinoside-8-C-hexoside (isomer 3)	41.6	C_26_H_28_O_14_	563.1400	−	+	[28,29]
sinapic acid	42.6	C_11_H_12_O_5_	223.0609	+	−	[29,47]
pinosylvin (double glycosylation)	44.8	C_26_H_32_O_12_	535.1844	+	+	[29]
glycosylated and acetylated 3, 4, 5′-trihydroxy-3, 7-dimethylflavone	46.4	C_25_H_26_O_13_	533.1295	−	+	[25,28,29]
apigenin-6-C-beta-galactosyl-8-C-beta-glucosyl-*O*-glucuronopyranoside (isomer 1)	51.7	C_33_H_38_O_21_	769.2009	+	+	[29]
apigenin-6-C-beta-galactosyl-8-C-beta-glucosyl-*O*-glucuronopyranoside (isomer 2)	52.9	C_33_H_38_O_21_	769.1977	+	+	[29]
5, 7, 4′-trihydroxy-3, 5′-dimethoxy-flavone (tricin)	69.3	C_17_H_14_O_7_	329.0687	−	+	[28,29]
formononetin (glycosylated and methylated)	70.9	C_23_H_24_O_9_	443.1379	+	+	[29,48]

* Rt, retention time; (+), presence; (−) absence.

**Table 4:** Estimated concentration of phenolics (mg/100 g DW) of durum wheat soluble extracts obtained under optimized conditions.

Compound	Breeding Line 5	Breeding Line 98
*p*-coumaric acid	nd	0.25
ferulic acid	2.69	4.18
total quantified phenolic acids	2.69	4.43
pinosylvin (double glycosylation) *	0.43	0.17
apigenin-6-C-arabinoside-8-C-hexoside *	8.38	4.13
apigenin-6-C-beta-galactosyl-8-C-beta-glucosyl-*O*-glucuronopyranoside *	2.44	3.43
total quantified flavonoids	11.26	7.73

Data represent mean values for each sample (*n* = 3). * Compounds quantified as catechin equivalents; Abbreviations are: nd, not detected; DW, dry weight.

Several studies addressing the phenolic profile of wheat were revised to discuss our findings. Dinelli et al. [29] reported the presence of various flavonoids in durum wheat and demonstrated that specific phenolic acids (e.g., *p*-coumaric acid) were not present in all varieties, which also agrees with our study. Di Loreto at al. [46] suggested possible differences between biosynthetic pathway of secondary metabolites among durum wheat genotypes. However, these authors only evaluated the presence of phenolic acids. In addition, according to Leoncini et al. [25], the total flavonoid content correlated with Ferric reducing antioxidant power (FRAP), but not with DPPH thus showing that the antioxidant properties of flavonoids from durum wheat remains to be completely clarified. These authors also identified seven flavone-C-glycoside and two phenolic acids in the free fraction of common wheat varieties, but they did not quantify any of them. Therefore, by reporting the quantities of these phenolics, the present work shed some light on the contribution of flavone-C-glycoside and phenolic acids in the potential bioactivity of durum wheat, as discussed in the following sections.

### 2.3. Antiradical Activity and Reducing Power

An editorial by Harnly [49] has put in check the validity of colorimetric methods to screen the antioxidant activity of polyphenols. In contrast, in a follow-up report [50] several experts have clarified that “for bioactive purposes, in vivo models are required or, at the very least, methods that employ distinct mechanisms of action (i.e., single electron transfer (SET), transition metal chelating ability, and hydrogen atom transfer (HAT))”. More recently, another study by members of our research group [51] demonstrated that FRAP and Oxygen Radical Absorbance Capacity (ORAC) values of phenolic extracts containing phenolic acids and flavonoids could anticipate the reduction of the activation of NF-κB using LPS-activated RAW 264.7 macrophages. Likewise, the highest ability of phenolic extracts in scavenging ABTS radical cation (2,2′-azino-bis (3-ethylbenzothiazoline-6-sulfonic acid) diammonium) and DPPH radical resulted in higher capacity in preventing the activation of NF-κB in LPS-activated RAW 264.7 exposed to flavonoids [52].

A recent study [53] demonstrated that phenolic acids inhibited thiobarbituric acid reactive substances (TBARS) production in a concentration-dependent manner in rat brain homogenate. The compound chlorogenic acid had a higher inhibitory ability (IC_50_ = 32.61 μM) compared to that of ferulic acid (IC_50_ = 81.38 μM), and a similar trend was found against DPPH radical. Therefore, the mentioned studies [51,52,53] lend support on the usefulness of colorimetric methods in the screening of potential biological properties of phenolic bioactives. In addition, a recent contribution improved the DPPH assay to overcome potential interferences of the tested material [54], also supporting the ongoing importance of colorimetric methods. Finally, Harnly [49] suggested that nonspecific analytical methods should be avoided as much as possible and researchers should focus on the analysis of specific compounds, when and where possible. Therefore, due to new identification tools (e.g., LC-MS) and the increasing evidence supporting the high contribution of flavonoids in durum wheat, the present study combines colorimetric methods and LC–ESI-QTOF-MS to better understand the chemistry involved in future health claims of these feedstocks.

Breeding line 98 exhibited the highest antiradical activity (13.1 to 77.1% higher), regardless of the method employed Table 5. The same trend was observed for FRAP (up to 60% higher). Interestingly, this lends support to the TPC of phenolic extracts obtained in any solvent system, which ranged from 24.57 to 166.04 mg GAE/100 g DW and from 21.73 to 134.24 mg GAE/100 g DW for breeding lines 98 and 5, respectively Table 1. Therefore, as suggested by experts in the field [50], we confirmed the greatest antioxidant potential of breeding line 98 by distinct mechanisms of action such as single electron transfer, hydrogen atom transfer, also complementing the drawbacks of DPPH and ABTS assays by testing the phenolics against reactive oxygen species (ROS) and ferric ions. Considering many publications reviewed during the course of this study, only one research team used acetone/water [55]. However, the authors did not use any optimization technique and employed a different proportion (4:1, *v/v*). In addition, a different species of wheat (common wheat (*Triticum aestivum*) varieties) was tested. Therefore, for the reasons given, comparison of the results would not be appropriate.

The highest contribution of derivatives of apigenin in both samples (63.5 to 80.7%) indicated that flavonoids could render the most significant contribution to the antioxidant activity of durum wheat. However, structure-activity relationships may be more relevant than the concentration of specific compounds [56,57]. In fact, the higher biological activity of flavonoids (e.g., isoflavones) as aglycones compared to that of their corresponding conjugates has been extensively discussed [52,58,59]. In contrast, although several authors have dealt with the phenolic profile of durum wheat, a proper discussion on the antioxidant properties of the identified flavonoids in this feedstock is lacking. The results of the present contribution do not support a positive influence on the antiradical properties and reducing power of durum wheat flavonoids, which were all present in their conjugated form. Instead, the greater concentration of phenolic acids in breeding line 98 (64.7%) is quite similar to the higher antiradical activity toward peroxyl radical (77.1%) and reducing power (60%), thus confirming the dose-dependent effect of phenolic acids, but not of wheat flavonoids.

Oxidizing agents damage deoxyribose and DNA. Likewise, copper bioavailability is a predictor of early atherosclerosis [60]. Phenolics from durum wheat and its processing by-products exhibited protection towards ROS-induced DNA damage and copper-induced oxidation of human low-density lipoprotein (LDL) cholesterol in vitro [1]. Likewise, ferulic acid, the main hydroxycinnamic acid quantified in the present study, was found as the most active compound toward hydrogen peroxide- and UV-induced DNA damage compared to that of caffeic, chlorogenic, cinnamic, gallic, *p*-hydroxybenzoic, protocatechuic, rosmarinic, syringic, and vanillic acids [61]. The protective effect of ferulic acid against copper-mediated human LDL peroxidation has also been reported [62]. Furthermore, *p*-coumaric acid has been found to be positively and significantly associated with the two biological model systems (ROS-induced DNA damage and inhibition of oxidation of LDL-cholesterol) [63]. DNA damage and repair are important mechanisms related to development of cancer [64] while LDL-cholesterol oxidation is an important biomarker related to development of coronary heart disease [16]. Therefore, due to potential effects against the aforementioned diseases, in addition to the lack of evidence with respect to the contribution of conjugated flavonoids to the antioxidant properties of durum wheat reported here, future efforts should be directed to developing high-phenolic acid durum wheat cultivars and/or, possibly, with improved contents of flavonoids in the aglycone form.

### 2.4. Inhibition of Pancreatic Lipase and Alpha-Glucosidase Activity

Polyphenols bind proteins due to the formation of hydrogen bonds or addition of nucleophiles to oxidized phenolics as quinones and specific methods have been developed for discovering new enzyme inhibitors aiming at designing new functional foods and/or nutraceuticals [65]. Accordingly, several sources of phenolic compounds have been tested for their inhibitory activity toward alpha-glucosidase [66,67]. The antidiabetic effect of natural compounds has been in the spotlight of various researchers [68,69]. Alpha-glucosidase, an enzyme found in the brush border of the small intestine, inhibits the absorption of carbohydrates and may suppress the postprandial blood glucose and insulin levels, while pancreatic lipase is a key enzyme regulating the absorption of triacylglycerols [19]. As mentioned earlier, the higher biological activity of phenolic compounds as aglycones compared to that of their corresponding conjugates has been extensively discussed [52,58,59] which may induce one to suggest that all conjugated phenolics will exhibit a low inhibitory effect. However, after employing bioactivity-guided fractionation methods, Sun et al. [70] isolated and identified 6-*O*-D-glycosides (e.g., 6-*O*-*p*-*trans*-coumaroyl-D-glucopyranosides) as potent alpha-glucosidase inhibitors thus showing that one should not assume that all phenolic conjugates show negligible inhibition toward digestive enzymes and that this subject is worthy of investigation.

In this study, a very small alpha-glucosidase inhibitory activity was found for the phenolic extracts obtained under optimized conditions Table 5. However, although no evidence was found for significant anti-diabetic potential, the chemical results lend support to the trend found in the experiment screening of the ability from the same extracts against pancreatic lipase activity. While the alpha-glucosidase inhibitory effect of breeding line 98 was 2.7-fold higher than that of breeding line 5, the first one showed 43.5% greater lipase inhibition than the latter genotype Table 5, exhibiting a significant inhibitory activity that ranged from 40.2 to 57.7%.

Durum wheat is mainly used in the pasta industry. Spaghetti, and lasagna, among other pasta products, are usually prepared with different lipid sources (e.g., olive oil, milk, meat, and their products). According to the present results, it is possible to suggest that due to its higher inhibitory activity toward pancreatic lipase, high-phenolic acid durum wheat varieties (e.g., breeding line 98), but not high in derivatives of apigenin, may decrease the absorption of triacylglycerols present in the ingredients of pasta. However, confirmation in vivo is deemed necessary.

## 3. Materials and Methods

### 3.1. Samples, Chemicals, and Solvents

#### 3.1.1. Plant Materials

Advanced breeding lines (lines 5 and 98) of durum wheat were obtained from the International Maize and Wheat Improvement Center (CIMMYT, Obregón, Sonora, Mexico). In accordance with their performance under field conditions as well as quality evaluations carried out by the above-mentioned institution in Ciudad Obregón, Sonora, Mexico, line 5 and line 98 are characterized as strong and weak gluten genotypes, respectively. Grain quality tests supporting their respective classification have been addressed by our research group and reported elsewhere [71].

#### 3.1.2. Chemicals and Solvents

Total phenolic content (TPC): Folin-Ciocalteu reagent was purchased from Dinâmica (Dinâmica Química Contemporânea, Diadema, SP). Sodium carbonate was obtained from Synth (Synth, Diadema, SP, Brazil) and gallic acid was procured from Sigma–Aldrich (St. Louis, MO, USA). Phenolic profile: acetonitrile and methanol were procured from J.T. Baker (Phillipsburg, NJ, USA); formic acid was purchased from Tedia (Fairfield, OH, USA); water was obtained from a Millipore Milli-Q System (Millipore SAS, Molsheim, France). ABTS radical cation scavenging activity: ethanol was acquired from Synth (Diadema, SP, Brazil) while (±)-6-hydroxy-2,5,7,8-tetramethylchroman-2-carboxylic acid (trolox), dibasic potassium phosphate, monobasic potassium phosphate, and 2,2′-azino-bis (3-ethylbenzothiazoline-6-sulfonic acid) diammonium salt (ABTS) were purchased from Sigma-Aldrich (St. Louis, MO, USA). DPPH radical scavenging activity: 1,1-diphenyl-2-picrylhydrazyl (DPPH) was obtained from Sigma-Aldrich (St. Louis, MO, USA), and methanol was purchased from J.T. Baker (Phillipsburg, NJ, USA). Oxygen radical absorbance capacity: dibasic potassium phosphate, fluorescein sodium salt, and 2,2-azobis(2-methylpropionamidine) dihydrochloride (AAPH), and (±)-6-hydroxy-2,5,7,8-tetramethylchroman-2-carboxylic acid (trolox) were purchased from Sigma-Aldrich (St. Louis, MO, USA). Ferric reducing antioxidant power (FRAP): Potassium chloride and ethanol were purchased from (Synth, Diadema, SP, Brazil) while 2,4,6-tripyridyl-S-triazine (TPTZ) was acquired from Sigma-Aldrich (St. Louis, MO, USA). Inhibition of pancreatic lipase activity: Lipase from porcine pancreas, 4-methylumbelliferyl oleate, and trizma^®^ hydrochloride were purchased from Sigma-Aldrich (St. Louis, MO, USA), while sodium citrate was procured from Synth (Synth, Diadema, SP, Brazil). Inhibition of alpha-glucosidase: *p*-nitrophenyl α-d-glucopyranoside (*p*-NPG) and alpha-glucosidase were provided by Sigma-Aldrich (St. Louis, MO, USA). HPLC standards were purchased from Sigma-Aldrich (St. Louis, MO, USA). The remaining chemicals and solvents were of analytical or chromatographic grade and were used as received.

#### 3.1.3. Extraction Procedure

The samples were ground using an analytical mill (Model A11 B S32, IKA^®^, Germany). Ground samples were defatted with hexane using a solid/solvent ratio of 1:5 (*w/v*) by centrifugation at 5000× *g* using an Eppendorf 5810 R centrifuge (Eppendorf, Hamburg, Germany). The hexane was removed by vacuum filtration and the process was repeated three more times. Furthermore, the defatted samples were placed in an oven at 40 °C for 24 h to remove any remaining solvent. The extracts were obtained from defatted samples using acetone, water, and methanol as well as their binary or ternary mixtures. In summary, defatted samples (2 g) were mixed with 10 mL of solvent in concentrations summarized in Table 1. The mixture was placed in a water bath shaker (model Dubnoff SL-157, Piracicaba, SP, Brazil) at 30 °C for 20 min. The contents were centrifuged at 5000× *g*. The supernatants were collected and used for further analysis.

#### 3.1.4. Total Phenolic Content (TPC)

TPC was estimated following the Folin-Ciocalteu method [72] with minor changes as described by Salvador et al. [73]. The extraction of soluble phenolics of whole durum wheat was carried out gyratory water bath shaker at 30 °C for 20 min [74]. Phenolic extracts obtained under the conditions shown in Table 1 (20 μL) and and Folin–Ciocalteu (10% *v/v*, 100 μL) were pipetted into each microplate well. Sodium carbonate solution (7.5% *v/v*, 75 μL) was pipetted into the wells after 5 min of reaction and the mixture was stirred using a microplate reader (Molecular Devices, LLC, Sunnyvale, CA, USA) and the absorbance was read at 734 nm using the same equipment after 40 min of reaction in the dark. The results were expressed as gallic acid equivalents (GAE)/100 g of sample (mg GAE/100 g).

#### 3.1.5. LC–ESI-QTOF-MS Analysis

Phenolic extracts (20 µL) obtained under the optimal extraction conditions (aqueous acetone 50%, *v/v*) were used for LC–ESI-QTOF-MS analysis. High resolution mass spectrometry analysis was carried out using a Shimadzu chromotagraph (Shimadzu Co., Kyoto, Japan) connected to an LC-30 AD quaternary pump, SIL-30 AC self-injector, and photodiode array detector. The chromatographic separation was realized on a Phenomenex Luna c18 column (4.3 × 250 × 5 µm). The binary mobile stage corresponded to a blend of two solvents: (A) water/formic acid (99.9:0.1, *v/v*) and (B) acetonitrile/formic acid (99.9:0.1, *v/v*). The flow rate was 1.0 mL/min, and the gradient was initiated with 5% of solvent B, augmenting to 7% in 7 min, 20% in 50 min, 45% in 70 min, 100% in 85 until 90 min, restarting to 5% in 100 min and ending the run in 110 min. The operating parameters were programmed using the following factors: temperature, 200 °C, HV, 4500, nebulizer 2 bar, and dry gas, 8 L/min. Prior to this analysis, an external calibration was carried out to identify the accuracy (precision) of the masses recorded/scored which, according to authentic standards, ranged from 5.4085 to 9.1437 ppb. Data analysis was done by employing MAXIS 3G software (Bruker Daltonics, version 4.3) interfaced through an electrospray interface (ESI-negative mode) with a nominal resolution of 60,000 *m/z*. The identification of the compounds was made by comparing the exact masses, literature data and, available phenolic standards. The scan range was set from *m/z* 50 to 800 to obtain the total ion current (TIC) chromatogram while the extracted ion current (EIC) chromatogram [*m/z* at 163 for *p*-coumaric acid, *m/z* at 193 for ferulic acid, *m/z* at 535 for pinosylvin, *m/z* at 563 for apigenin-6-C-arabinoside-8-C-hexoside, and *m/z* at 769 for apigenin-6-C-beta-galactosyl-8-C-beta-glucosyl-*O*-glucuronopyranoside] was used for quantification purposes. *p*-Coumaric acid, ferulic acid, and catechin were dissolved in water/formic acid (99.9:0.1, *v/v*), the same solvent system was used as mobile phase A, to prepare the calibration curves (5–250 mg/L). The regression coefficients had r^2^ ranging from 0.9990 to 0.9999, while the limits of detection and quantification ranged from 80 to 90 ng/g and from 270 to 300 ng/g, respectively.

#### 3.1.6. ABTS Radical Cation Scavenging Activity

The ABTS radical cation scavenging activity was evaluated according to Salvador et al. [73]. ABTS radical cation was generated by the reaction of 7 mmol/L ABTS (5 mL) with 140 mmol/L potassium persulfate (88 µL). The blend was incubated at 25 °C for 16 h in the dark. Potassium phosphate buffer (75 mmol/L, pH 7.4) was employed to dilute the stock solution to an absorbance of 0.700 ± at 734 nm. Phenolic extracts obtained under the optimal extraction conditions (aqueous acetone 50%, *v/v*) were used for the experiments. Aliquots of the samples (20 µL) and ABTS radical cation (220 µL) were then placed into microplate wells and blended. The absorbance was studied at 734 mm passed 6 min of reaction using a microplate reader SpectraMax^®^ M3 (Molecular Devices LLC, Sunnyvale, CA, USA). Potassium phosphate buffer was used as blank. The results were expressed as trolox equivalents (TE)/100 g of sample (µmol TE/100 g).

#### 3.1.7. DPPH Radical Scavenging Activity

The DPPH radical scavenging activity was evaluated according to Salvador et al. [73]. DPPH radical (180 µmol/L) was prepared in methanol. Phenolic extracts obtained under the optimal extraction conditions (aqueous acetone 50%, *v/v*) were used for the experiments. Aliquots of the samples (20 μL) and DPPH radical (200 μL) were transferred to microplate wells and mixed. The absorbance was read at 515 nm after 30 min of reaction using a microplate reader (SpectraMax M5; Molecular Devices Corp., Sunnyvale, CA, USA). The results were expressed as trolox equivalents (TE)/100 g of sample (µmol TE/100 g).

#### 3.1.8. Oxygen Radical Absorbance Capacity (ORAC)

Phenolic extracts obtained under the optimal extraction conditions (aqueous acetone 50%, *v/v*) were used for the experiments. The ORAC test was used to evaluate the ability of phenolic extracts in scavenging peroxyl radicals. The adjusted protocol was outlined in de Camargo et al. [51]. Aliquots of the phenolic extracts (30 μL), 508.25 nmol/L fluorescein (60 μL), and 76 mmol/L AAPH (110 μL) were mixed and placed into microplates. Potassium phosphate buffer (75 mmol/L, pH 7.4) was used to dilute the solutions and also used as a blank. Eventually, the reaction was performed at 37 °C, and the reads were scored every 2 min over a 2 h time period, at excitation and emission wavelengths of 485 and 528 nm, respectively, employing a microplate reader SpectraMax^®^ M3 (Molecular Devices LLC, Sunnyvale, CA, USA). The outcomes of each defatted sample were asserted and reported as micromoles of trolox equivalents (TE)/100 g of sample (µmol TE/100 g).

#### 3.1.9. Ferric Reducing Antioxidant Power (FRAP)

FRAP was studied based on the protocol reported in de Camargo et al. [51]. The FRAP reagent was obtained by combining 2.5 mL FeCl_3_·6H_2_O solution, 2.5 mL TPTZ solution, and 25 mL 300 mmol/L acetate buffer (pH 3.6). Phenolic extracts obtained under optimal extraction conditions (aqueous acetone 50%, *v/v*) were used for the experiments. Aliquots of the samples (20 μL) were blended with distilled water (30 μL) and FRAP reagent (200 μL). The mixture was maintained at 37 °C for 8 min. The measurements were carried out at 595 nm in a microplate reader SpectraMax^®^ M3 (Molecular Devices LLC, Sunnyvale, CA, USA). The results were expressed as micromoles of Fe^2+^/100 g of sample (µmol Fe^2+^/100 g).

#### 3.1.10. Inhibition of Pancreatic Lipase

Phenolic extracts obtained under optimal extraction conditions (aqueous acetone 50%, *v/v*) were used for the experiments. The inhibitory activity of phenolics from durum wheat against pancreatic lipase was evaluated using 4-methylumbelliferyl oleate (4-MU oleate) as a substrate as described by Kurihara et al. [75]. In the presence of pancreatic lipase, 4-methylumbelliferone and oleic acid are liberated from 4-MU oleate. The compound 4-methylumbelliferone is a chromogenic substance used for quantification purposes. 4-MU oleate (0.1 mM) and type II crude porcine pancreatic lipase (50 U/mL) were prepared in a buffer solution consisting of 13 mM Tris–HCl, 150 mM NaCl, and 1.3 mM CaCl_2_ (pH 8.0). Phenolic extracts (25 μL) were mixed with pancreatic lipase (25 μL) and 4-MU oleate (50 μL) and incubated in a microplate reader (SpectraMax M5; Molecular Devices Corp., Sunnyvale, CA, USA). To stop the reaction, an aliquot (0.1 mL) of sodium citrate (0.1 M, pH 4.2) was added to the mixture after 30 min of incubation at 37 °C. The measurements were recorded at excitation and emission wavelengths of 355 and 460 nm, respectively. The control was a mix of all solutions, except for the phenolic extract. The percentage of inhibition activity was quantified using the equation as follows:

Pancreatic lipase inhibition (%) = [(Abs_control_ − Abs_sample_)/(Abs_control_)] × 100 (3)

#### 3.1.11. Inhibition of Alpha-Glucosidase

Phenolic extracts obtained under optimal extraction conditions (aqueous acetone 50%, *v/v*) were used for the experiments. The inhibitory activity of phenolics from durum wheat against alpha-glucosidase was evaluated using *p*-nitrophenyl α-d-glucopyranoside (*p*-NPG) as a substrate as described elsewhere [76,77]. In the presence of alpha-glucosidase, *p*-NPG is hydrolyzed thus generating glucose and *p*-nitrophenol. The *p*-NPG (5 mM) and alpha-glucosidase [5 g in 10 mL of saline (0.9% *w/v*)] were prepared in a 0.1 M phosphate buffer (pH 6.8). Phenolic extracts (50 μL) were mixed with alpha-glucosidase (100 μL). After 10 min of incubation at 37 °C in a microplate reader (SpectraMax M5; Molecular Devices Corp., Sunnyvale, CA, USA), the *p*-NPG solution (50 μL) was added and incubated at the same temperature for 5 min. An aliquot (100 μL) of 0.1 M calcium carbonate was added to stop the reaction and the absorbance was read at 405 nm. A control (devoid of phenolic extracts) was prepared and subjected to the same procedures described before. The percentage of inhibition of activity was calculated using the following equation:

Alpha-glucosidase inhibition (%) = [(Abs_control_ − Abs_sample_)/(Abs_control_)] × 100 (4)

#### 3.1.12. Experimental Design and Statistical Analysis

The simplex-centroid mixture design was used to investigate the effect of different solvents (x_1,_ x_2,_ x_3_), where x_1_ is acetone, x_2_ is water, and x_3_ is methanol. The experimental design Table 1 was composed of seven solvent systems: three individual solvents (1, 0, 0), (0, 1, 0), (0, 0, 1) and three binary mixtures (0.5, 0.5, 0), (0.5, 0, 0.5), (0, 0.5, 0.5). The extraction at the central point (or centroid) represents an equal contribution of all three solvents (0.333, 0.333, 0.333). Runs 7–9, representing the extraction in three replicates, allow estimating the variation in the responses at the central point and provides a basis for the lack-of-fit test [78,79]. The response function was the TPC, which was evaluated in two different advanced breeding lines.

y_1_ and y_2_ stems from the TPC of Breeding Line 98 and Breeding Line 5, respectively. Mathematical models for the mixture design used was special cubic model:

$$y=\sum_{i=1}^{\;q}b_{i}^{*}x_{i}+\sum_{i<j}^{\;q}\sum_{j}^{\;q}b_{ij}^{*}x_{i}x_{j}+\sum_{i<j}^{\;q}\sum_{j<k}^{\;q}\sum_{k}^{\;q}b_{ijk}^{*}x_{i}x_{j}x_{k}+\ldots +b_{12\ldots q}^{*}x_{1}x_{2}\ldots x_{q}$$

where *y* = estimated response; b^*^ = estimated coefficient by least squares method, *x_i_* = dependent variable, with 1 > *x_i_* > 0 and ∑ *_xi_* = 1. The b^*^_i_ parameter is the linear coefficient that is associated with the pure component *i*; b^*^_ij_ is the quadratic coefficient of binary interaction for components *i* and *j*; b^*^_ijk_ corresponds to the cubic coefficient of ternary interaction for components *i, j* and *k* [80].

## 4. Conclusions

The highest total phenolic contents (TPC) were found using aqueous acetone (50%, *v/v*). Consequently, the use of aqueous methanol or aqueous ethanol, which are the most commonly used thus far, leads to underestimation of TPC. As for the potential bioactivity, breeding line 98 exhibited the highest antiradical activity toward peroxyl radical. The same trend was observed with respect to the reducing power (FRAP) and the inhibitory activity toward pancreatic lipase and alpha-glucosidase, although, considering the latter one, no evidence was found for significant anti-diabetic potential. Therefore, the potential bioactivity was found to be in accordance with the TPC. Most importantly, the concentrations of phenolic compounds obtained by LC–ESI-QTOF-MS do not support the contribution of flavonoids in the in vitro models. Instead, despite their lower concentration compared to that of flavonoids, structure-activity relationships still suggest the crucial role of phenolic acids of durum wheat in the manufacture of functional foods and/or ingredients. Finally, the current study discourage breeding programs as well as animal and/or human trials prospecting the effect of durum wheat flavonoids on oxidative stress and absorption of triacylglycerols.

## Figures and Tables

**Table 1:** Simplex-centroid design and response functions.

Extraction *	Solvent Mixture			TPC (mg GAE/100 g DW)		TPC Yield (%) **	
	Acetone (x_1_)	Water (x_2_)	Methanol (x_3_)	Breeding Line 98	Breeding Line 5	Breeding Line 98	Breeding Line 5
1	1	0	0	24.57	21.73	15	16
2	0	0	1	50.90	36.99	31	28
3	0	1	0	77.76	73.99	47	55
4	0.5	0	0.5	48.67	34.96	29	26
5	0	0.5	0.5	120.35	104.28	72	78
6	0.5	0.5	0	166.04	134.24	100	100
7	0.333	0.333	0.333	135.22	118.53	81	88
8	0.333	0.333	0.333	134.21	123.97	81	92
9	0.333	0.333	0.333	126.07	129.37	76	96

TPC, total phenolic content; GAE, gallic acid equivalents; DW, dry weight. * Runs 7–9 allow estimating the variation in the responses at the central point and provides a basis for the lack-of-fit test (*n* = 3). ** 100% yield was assigned to the extraction showing the highest TPC.

**Table 2:** ANOVA of the special cubic models adjusted to the experimental data from simplex-centroid design.

Source	Breeding Line 98				Breeding Line 5			
	SS	MS	*F*-Value	*p*	SS	MS	*F*-Value	*p*
Model	18,915.89	4728.97	125.23	0.0002	16,242.52	3248.50	122.20	0.0012
Total error	151.05	37.76			79.75	26.58		
* Lack of Fit	100.71	50.35	2.00	0.3333	20.93	20.93	0.71	0.4877
* Pure error	50.34	25.17			58.82	29.41		
Total adjusted	19,066.94	2383.37			16,322.27	2040.28		
*R* ^2^	0.99				0.99			
Adjusted *R*^2^	0.98				0.99			

SS: Sum of squares; MS: Mean Squares. * Lack of fit and pure error are components of total error.

**Table 5:** Radical scavenging activity, reducing power, lipase and alpha-glucosidase inhibitory activity of phenolic extracts obtained under optimized conditions *.

Assay	Breeding Line 5	Breeding Line 98
ABTS (µmol TE/100 g DW)	378.1 ± 13.7 b	493.5 ± 23.8 a
DPPH (µmol TE/100 g DW)	108.3 ± 2.5 b	122.5 ± 3.0 a
ORAC (µmol TE/100 g DW)	438.0 ± 40.1 b	775.5 ± 61.6 a
FRAP (µmol Fe^2+^/100 g DW)	3.00 ± 0.0 b	4.80 ± 0.2 a
Inhibition of lipase activity (%)	40.2 ± 2.3 b	57.7 ± 7.2 a
Inhibition of alpha-glucosidase activity (%)	2.10 ± 0.0 b	5.65 ± 0.9 a

* Values expressed as means of triplicate determinations. TE, trolox equivalent; ABTS, 2, 2′-azino-bis (3-ethylbenzothiazoline-6-sulfonic acid) diammonium; DPPH, 1, 1-diphenyl-2-picrylhydrazyl; ORAC, oxygen radical absorbance capacity; FRAP, ferric reducing antioxidant power. Means with the same letters within a row are not significantly different according to Tukey’s multiple test (*p* > 0.05).

## Data Availability

The data presented in this study are available on request from the corresponding author.

## Sample Availability

Samples of the compounds are not available from the authors.
